# Preeclampsia and Retinopathy of Prematurity in Very-Low-Birth-Weight Infants: A Population-Based Study

**DOI:** 10.1371/journal.pone.0143248

**Published:** 2015-11-20

**Authors:** Hsin-Chung Huang, Hwai-I Yang, Hung-Chieh Chou, Chien-Yi Chen, Wu-Shiun Hsieh, Kuo-Inn Tsou, Po-Nien Tsao, Taiwan Premature Infant Developmental Collaborative Study Group

**Affiliations:** 1 Department of Pediatrics, Taipei City Hospital, Heping FuYou Branch, Taipei, Taiwan; 2 Genomics Research Center, Academia Sinica, Taipei, Taiwan; 3 Graduate Institute of Clinical Medicine, National Yang-Ming University, Taipei, Taiwan; 4 Department of Pediatrics, National Taiwan University Hospital, National Taiwan University College of Medicine, Taipei, Taiwan; 5 Department of Pediatrics, Catholic Tien Hospital, and College of Medicine, Fu Jen Catholic University, Taipei, Taiwan; 6 Research Center for Developmental Biology and Regenerative Medicine, National Taiwan University, Taipei, Taiwan; University of Utah (Salt Lake City), UNITED STATES

## Abstract

Preeclampsia and retinopathy of prematurity (ROP) are associated with impaired angiogenesis. Previous studies on the relationship between preeclampsia and ROP have produced conflicting results. The goal of this study was to evaluate the association between maternal preeclampsia and ROP using a large population-based cohort of very-low-birth-weight (VLBW) infants from 21 neonatal departments registered in the database of the Premature Baby Foundation of Taiwan. Multivariable logistic regression analysis was used to estimate the adjusted odds ratios (OR) and 95% confidence intervals (CI) for preeclampsia with reference to ROP and severe ROP. A total of 5,718 VLBW infants (844 cases with maternal preeclampsia) were included for analysis. The overall incidences of mild and severe ROP were 36.0% and 12.2%, respectively. Univariable analysis showed lower GA and lower birth weight, vaginal delivery, non-SGA, RDS, PDA, sepsis, transfusion, and absence of maternal preeclampsia to be associated with mild and severe ROP development. However, OR (95% CI) adjusted for the variables that were significant according to univariable analysis showed the risks of developing any-stage ROP and severe ROP for maternal preeclampsia to be 1.00 (0.84–1.20) and 0.89 (0.63–1.25), respectively. The results remained unchanged in stratified analyses according to SGA status. Our data showed that maternal preeclampsia was not associated with the subsequent development of any stage or severe ROP in VLBW infants.

## Introduction

Retinopathy of prematurity (ROP), a disease associated with abnormal retinal vascular development in preterm infants [1], occurs frequently among very preterm infants [2, 3]. With the improved survival of this population due to advances in neonatal care, ROP has become a leading cause of childhood blindness worldwide [4]. Several risk factors, including small gestational age, low birth weight, and postnatal oxygen therapy, are known to be associated with the development of ROP [5, 6]. Preeclampsia causes maternal and fetal morbidity and is also a leading cause of preterm delivery of VLBW infants [7, 8]. Dysregulation of circulating antiangiogenic factors plays an important role in the pathogenesis of both preeclampsia and ROP [9, 10]. Previous studies have analyzed the relationship between maternal preeclampsia and ROP in preterm infants; however, the results have been inconsistent and conflicting due in part to the relatively small sample sizes of the studies [11–17].

Recently, 3 large-scale studies analyzing the association between preeclampsia and ROP have produced inconsistent results. Yu et al. found an association between preeclampsia and a significantly reduced risk of ROP in preterm infants.[18] However, they did not adjust for SGA, which was more common in the maternal preeclampsia group and was significantly associated with ROP [18–20]. Araz-Ersan et al. demonstrated that maternal preeclampsia was associated with decreased incidence of progression to severe ROP, but not the onset of ROP. [21]. In addition, they did not adjust their findings for other confounding factors. Lee et al. reported that maternal preeclampsia was not associated with any stage of ROP in Extremely Low Gestational Age Newborns. However, extremely preterm infants born to mothers with preeclampsia, which is associated with neonatal hyperoxemia and bacterial infection, have an increased risk of severe ROP [22]. Because of the numerous discrepancies in previous studies, we examined the association between maternal preeclampsia and ROP in a large population-based cohort of very-low-birth-weight (VLBW) infants. The goal was to demonstrate the independent association between the two variables. We also performed subgroup analysis based on SGA status, which was shown in previous studies to be strongly associated with both maternal preeclampsia and ROP.

## Methods

### Study subjects

A total of 8,652 VLBW infants were registered in the database of the Premature Baby Foundation of Taiwan between 1997 and 2006. All 21 NICUs in Taiwan participated in this project, making the data a truly population-based cohort. The data collected included prenatal, perinatal, and postnatal demographic and clinical variables. Patient information obtained by the database coordinator was cross-checked with our national birth registry. The exclusion criteria included GA above 36 weeks, congenital or chromosome anomalies, infants who died before the screening of ROP, and those whose ROP status was not available. The percentages of infants excluded because the ROP status was not available were 0.4% in the non-preeclampsia group and 10.9% in the preeclampsia group. There were no differences in GA, BW, incidence of transfusion requirement, or sepsis in the two groups that were excluded for analysis. In addition, infants with maternal chronic hypertension were also excluded. Preeclampsia was defined as a diastolic blood pressure of greater than 90 mm Hg accompanied by proteinuria of at least 1+ (30 mg per deciliter) on dipstick test or nondependent edema during pregnancy. The gestational age (GA) was dated by the last menstrual period or the date of embryo transfer for *in vitro* fertilization. The ROP status was determined by pediatric ophthalmologists in each hospital.

### Ethics Statement

Written informed consent was obtained from the parents or designated relatives of the infants in the study. The study was approved by the Institutional Review Board of National Taiwan University Hospital and the Joint Institutional Review Boards of Taipei City Hospital, Cathay General Hospital, Younghe Cardinal Tien Hospital, Shin Kong Wu Ho-Su Memorial Hospital, Taipei Veterans General Hospital, Taipei Chang Gung Memorial Hospital, Tri-Service General Hospital, Mackay Memorial Hospital, China Medical University Hospital, Chung Shan Medical University Hospital, Taichung Veterans General Hospital, Changhua Christian Hospital, National Cheng Kung University Hospital, Chi Mei Hospital, Sin-Lau Hospital, Kuo General Hospital, Chia-Yi Christian Hospital, Kaohsiung Veterans General Hospital, Kaohsiung Chang Gung Memorial Hospital, and Kaohsiung Medical University Chung-Ho Memorial Hospital.

### Outcome variables

Mild and Severe ROP were defined using the criteria of an international committee [23]. Severe ROP was defined as stage 3 ROP plus any disease, and stage 4 or 5 ROP [23]. Respiratory distress syndrome (RDS) was defined by clinical diagnosis and need for surfactant therapy. Necrotizing enterocolitis (NEC) was defined according to the criteria of Bell [24]. Small for gestational age (SGA) was defined as birth weight of less than the 10^th^ percentile for the gestational age [25]. Transfusion was defined as requiring PRBC transfusion. Sepsis was defined as clinical sepsis with proof of causative agent in the blood culture. PDA required treatment means PDA required indomethacin/ibuprofen treatment or surgical ligation.

### Statistical analysis

The chi-square test and Student’s *t*-test were used for comparing distributions of categorical variables and the continuous variables between groups, respectively. A multivariable logistic regression model was used to analyze the association between maternal preeclampsia and ROP risk, adjusted for variables that were found to be statistically significant by univariate analysis. Adjusted odds ratio (OR) and 95% confidence interval (CI) were derived to assess the magnitude of the association between various factors and ROP risk. We performed a similar analysis to evaluate the association between maternal preeclampsia and ROP in subgroups of different severities of ROP (mild ROP, severe ROP, ROP without therapy, and ROP requiring therapy). Statistically significant levels were determined by the 2-tailed test (*p*<0.05). The association between preeclampsia and ROP was further examined in subgroup analysis with stratification according to SGA status.

## Results

A total of 5,718 VLBW infants, including 844 (14.8%) cases with maternal preeclampsia, were included for analysis. The numbers (overall incidence) of mild and severe ROP were 2,087 (36.5%) and 698 (12.2%), respectively. Infants born to a mother with preeclampsia were more likely to have higher gestational age, higher maternal age, delivery via Cesarean section, female gender, singleton, higher Apgar score at 5 minutes, and small for gestational age (SGA). They were less likely to have respiratory distress syndrome (RDS), patent ductus arteriosus (PDA), sepsis, and transfusion. The incidence of ROP was significantly lower in infants with maternal preeclampsia than in those without maternal preeclampsia (41.4% vs. 50%) (Table 1).

**Table 1:** Demographic and clinical variables in infants born to mothers with or without preeclampsia.

Parameter	No preeclampsia N = 4874	Preeclampsia N = 844	P value
Gestational age	29.04 (2.66)	30.97 (2.44)	<0.0001
Birth weight	1161 (237)	1161 (245)	0.9702
Maternal age	29.60 (5.37)	31.54 (5.11)	<0.0001
Cesarean section^†^	2713 (56.0%)	774 (91.9%)	<0.0001
Male sex^‡^	2530 (52.1%)	388 (46.2%)	0.0017
SGA	1263 (25.9%)	619 (73.3%)	<0.0001
Singleton^§^	3538 (72.8%)	716 (85.2%)	<0.0001
Antenatal steroid ≥ 2 doses^‖^	1364 (32.4%)	217 (29.6%)	0.1286
RDS**	2213 (46.0%)	261 (31.7%)	<0.0001
Transfusion	3070 (63.0%)	424 (50.2%)	<0.0001
PDA requiring treatment^††^	1708 (35.2%)	209 (24.9%)	<0.0001
Sepsis^§§^	1220 (25.1%)	174 (20.7%)	0.0062
Apgar score at 5 min ≥7^‡‡^	3092 (67.5%)	614 (73.9%)	0.0003
ROP	2436 (50.0%)	349 (41.4%)	<0.0001
Days on IPPV	14.6 (26.3)	7.3 (15.3)	<0.001
Days on oxygen, CPAP, or IPPV	41.9 (38.3)	26.3 (29.3)	<0.001
Duration of hospitalization	73.8 (35.9)	61.8 (27.6)	<0.001

Data was presented as mean (SD) or number (%).

Abbreviations: SGA, small for gestational age; RDS, respiratory distress syndrome with surfactant treatment; PDA, patent ductus arteriosus.

^†^A total of 27 subjects were missing on this variable.

^‡^ A total of 17 subjects were missing on this variable.

^§^ A total of 20 subjects were missing on this variable.

^‖^A total of 774 subjects were missing on this variable.

**A total of 86 subjects were missing on this variable.

^††^A total of 24 subjects were missing on this variable.

^§§^A total of 26 subjects were missing on this variable.

^‡‡^A total of 309 subjects were missing on this variable.

Multivariable logistic analysis, which included preeclampsia, GA, birth weight (BW), Cesarean section, sex of baby, SGA, RDS, PDA, sepsis, transfusion, and Apgar score at 5 min as predictors, showed that the preeclampsia was not associated with all grade ROP (odds ratio (95% CI) of 1.00 (0.84–1.20)). GA (0.81 (0.77–0.85)), Cesarean section (0.84 (0.74–0.95)), SGA (1.25 (1.01–1.55)), Apgar score at 5 minutes (0.75 (0.65–0.86)), transfusion (1.26 (1.11–1.44)), PDA (1.44 (1.26–1.64)), and sepsis (0.84 (0.73–0.96)) were associated with ROP (Table 2). Multivariable logistic regression analysis of different ROP severity showed that preeclampsia was not associated with either mild ROP (1.03 (0.86–1.24)) or severe ROP (0.89 (0.63–1.25)). Only GA (0.67 (0.61–0.73)), BW (0.83 (0.77–0.90), Apgar score at 5 minutes (0.81 (0.66–1.00)), and PDA (1.28 (1.04–1.57)) were associated with severe ROP (Table 3).

**Table 2:** Multivariable-adjusted odds ratios of developing ROP (all grades) for various factors.

Parameter	Odds ratio (95% CI)	P value
Preeclampsia (yes vs. no)	1.00 (0.84–1.20)	0.9884
Gestational age (per week)	0.81 (0.77–0.85)	< .0001
Birth weight, per 100 grams	0.92 (0.88–0.97)	0.0005
Cesarean section (yes vs. no)	0.84 (0.74–0.95)	0.0064
Sex of baby (male vs. female)	1.09 (0.96–1.23)	0.1725
SGA (yes vs. no)	1.25 (1.01–1.55)	0.0407
Apgar score at 5 min (≥7 vs. <7)	0.75 (0.65–0.86)	< .0001
RDS (yes vs. no)	0.93 (0.81–1.07)	0.3222
Transfusion (yes vs. no)	1.26 (1.11–1.44)	0.0005
PDA (required treatment vs. no)	1.44 (1.26–1.64)	< .0001
Sepsis (yes vs. no)	0.84 (0.73–0.96)	0.0123

Abbreviations: SGA, small for gestational age; RDS, respiratory distress syndrome with surfactant treatment; PDA, patent ductus arteriosus.

**Table 3:** Multivariable-adjusted odds ratio of developing mild and severe ROP for various factors in polytomous logistic analysis.

	Mild ROP vs. no ROP		Severe ROP vs. no ROP	
Parameter	Odds ratio (95% CI)	P value	Odds ratio (95% CI)	P value
Preeclampsia (yes vs. no)	1.03 (0.86–1.24)	0.7504	0.89 (0.63–1.25)	0.5078
Gestational age (per week)	0.84 (0.80–0.89)	< .0001	0.67 (0.61–0.73)	< .0001
Birth weight, per 100 grams	0.95 (0.91–1.00)	0.0537	0.83 (0.77–0.90)	< .0001
Cesarean section (yes vs. no)	0.85 (0.74–0.97)	0.0158	0.83 (0.68–1.02)	0.082
Sex of baby (male vs. female)	1.09 (0.96–1.23)	0.2076	1.11 (0.91–1.35)	0.2959
SGA (yes vs. no)	1.19 (0.95–1.49)	0.1214	1.35 (0.95–1.93)	0.0979
Apgar score at 5 min (≥7 vs. <7)	0.74 (0.64–0.85)	< .0001	0.81 (0.66–1.00)	0.0449
RDS (yes vs. no)	0.88 (0.76–1.01)	0.0735	1.19 (0.96–1.49)	0.1192
Transfusion (yes vs. no)	1.32 (1.15–1.52)	< .0001	1.10 (0.88–1.38)	0.4014
PDA (yes vs. no)	1.48 (1.29–1.70)	< .0001	1.28 (1.04–1.57)	0.0177
Sepsis (yes vs. no)	0.81 (0.70–0.94)	0.0049	0.92 (0.75–1.14)	0.4607

Abbreviations: SGA, small for gestational age; RDS, respiratory distress syndrome with surfactant treatment; PDA, patent ductus arteriosus.

Since SGA was strongly associated with preeclampsia and ROP, we further performed subgroup multivariate-adjusted analysis with stratification according to SGA status. Maternal preeclampsia was not related with ROP in either the SGA group (0.98 (0.78–1.23), P = 0.8329) or the non-SGA group (1.06 (0.78–1.43), P = 0.7177). The risk factors of ROP included small GA, small BW, vaginal delivery, Apgar score at 5 minutes below 7, transfusion, PDA, and non-sepsis VLBW. In the SGA group, the risk factors of ROP, except for BW, Apgar score at 5 minutes below 7, and sepsis, were similar those in the non-SGA group (Table 4).

**Table 4:** Multivariable-adjusted odds ratios of developing ROP in stratified analysis according to SGA status.

	Non-SGA group		SGA group	
Parameter	Odds ratio (95% CI)	P value	Odds ratio (95% CI)	P value
Preeclampsia (yes vs. no)	1.06 (0.78–1.43)	0.7177	0.98 (0.78–1.23)	0.8329
Gestational age (per week)	0.84 (0.78–0.91)	< .0001	0.78 (0.72–0.84)	< .0001
Birth weight, per 100 grams	0.89 (0.83–0.95)	0.0002	0.96 (0.89–1.03)	0.2157
Cesarean section (yes vs. no)	0.85 (0.74–0.99)	0.035	0.76 (0.57–1.00)	0.0491
Sex of baby (male vs. female)	1.04 (0.90–1.20)	0.6153	1.24 (1.00–1.54)	0.0522
Apgar score at 5 min (≥7 vs. <7)	0.70 (0.59–0.82)	< .0001	0.91 (0.70–1.18)	0.4713
RDS (yes vs. no)	0.93 (0.79–1.09)	0.3479	0.96 (0.72–1.27)	0.7603
Transfusion (yes vs. no)	1.25 (1.07–1.47)	0.0055	1.32 (1.05–1.66)	0.0196
PDA (yes vs. no)	1.37 (1.18–1.60)	< .0001	1.62 (1.24–2.12)	0.0004
Sepsis (yes vs. no)	0.84 (0.72–0.99)	0.0421	0.83 (0.63–1.09)	0.1728

Abbreviations: SGA, small for gestational age; RDS, respiratory distress syndrome with surfactant treatment; PDA, patent ductus arteriosus.

Because of the known phenomenon that ROP occurs almost exclusively in infants < 34 weeks, we analyzed the data of 5,296 VLBW infants of < 34 weeks, including 717 (13.5%) with maternal preeclampsia. The numbers (incidence) of mild ROP and severe ROP were 2,006 (37.9%) and 692 (13.1%), respectively. The incidence of ROP was significantly decreased in infants with maternal preeclampsia (45% vs. 51.9%, P = 0.001). Again, multivariable analysis demonstrated that maternal preeclampsia was not associated with ROP (1.03 (0.85–1.25)), mild ROP (1.14 (0.85–1.54)), or severe ROP (1.00 (0.71–1.41)). Finally, when we focused on those extremely low birth weight (ELBW, birth weight below 1,000 gm) infants, multivariable polytomous analysis still showed that maternal preeclampsia was not associated with ROP (adjusted OR 0.89 (0.61–1.29)).

## Discussion

Our population-based large cohort study showed that maternal preeclampsia was not associated with ROP risk in VLBW infants. This finding is consistent with the study by Lee et al., which showed that preeclampsia itself was not associated with ROP in ELGANs [22]. In addition, our data also support the findings of other studies showing that lower GA, NSD, SGA, PDA, sepsis, transfusion, and lower Apgar score at 5 minutes are associated with ROP [12–19, 21, 22, 26–29].

Preeclampsia occurs in 2%-7% of pregnancies worldwide and results in fetal and maternal morbidity [8, 30]. Increasing circulating soluble Flt-1, a soluble form of vascular endothelial growth factor (VEGF) receptor-1, which can bind both VEGF and placental growth factor (PGF), plays an important role in the pathogenesis of preeclampsia [31–34]. VEGF signaling also plays a critical role in the pathogenesis of ROP, and anti-VEGF therapy has been shown to have significant benefits in ROP treatment [35, 36]. Several reports have produced inconsistent results on the association between maternal preeclampsia and ROP [11–17]. Recently, 2 large-scale studies reported that maternal preeclampsia significantly reduced the incidence of ROP in preterm infants [18, 21]. However, they did not adjust for SGA, which is strongly associated with both preeclampsia and ROP [18–20], as a confounder. In agreement with the studies by Yu et al. and Araz-Ersan et al., we found in this large multicenter study that VLBW infants delivered by mothers with preeclampsia had significantly lower incidence of ROP. However, after adjusting for confounding factors, including SGA and GA, we demonstrated that maternal preeclampsia was not associated with the risk of ROP in VLBW infants. Nonetheless, with the large sample size, we were able to do the subgroup analysis according to the SGA status, GA groups, or severity of ROP. Again, multivariate logistic regression analysis showed that maternal preeclampsia was not associated with ROP in either the SGA group or the non-SGA group, or in various GA strata, nor was maternal preeclampsia associated with either mild ROP or severe ROP. These findings suggest that although preterm infants born to mothers with preeclampsia have significantly lower incidence of ROP, maternal preeclampsia itself is not a protective factor, which is supported by a report from Lee et al. [22]. However, because potential bias such as small GA, low birth weight, and SGA may contribute to both preeclampsia and ROP, it is difficult to study an independent association between maternal preeclampsia and ROP.

The strength of our study is that it was a large multicenter population-based cohort study with several subgroup analyses, allowing us to assess the association between maternal preeclampsia and ROP. However, our study also had some limitations. First, some data of interest were unavailable (see tables). However, the large size of our database and the absence of differential misclassification would minimize these influences. Second, our cohort included only infants of birth weight below 1,500 gm, so data on larger infants born above 30 weeks’ gestation are not available, and thus infants born SGA are overrepresented in this group. However, we performed several subgroup analyses to minimize this bias.

## Conclusions

Although there was potential bias of a link between preeclampsia and ROP, our large population-based retrospective analysis found no association between these two diseases.
